# Verification and Validation of an Autotuning Proportional–Integral–Derivative Controller for Spatially Confined Magnetic Particle Hyperthermia

**DOI:** 10.1115/1.4070364

**Published:** 2025-12-22

**Authors:** Shreeniket Pawar, Hayden Carlton, Yash Sharad Lad, Lyndsey Werhane, Ma’Moun Abu-Ayyad, Preethi Korangath, Robert Ivkov, Anilchandra Attaluri

**Affiliations:** Department of Mechanical Engineering, School of Science, Engineering, and Technology, The Pennsylvania State University—Harrisburg, Middletown, PA 17057; Department of Radiation Oncology and Molecular Radiation Sciences, Johns Hopkins University School of Medicine, Baltimore, MD 21231; Department of Mechanical Engineering, School of Science, Engineering, and Technology, The Pennsylvania State University—Harrisburg, Middletown, PA 17057; Department of Radiation Oncology and Molecular Radiation Sciences, Johns Hopkins University School of Medicine, Baltimore, MD 21231; Department of Mechanical Engineering, School of Science, Engineering, and Technology, The Pennsylvania State University—Harrisburg, Middletown, PA 17057; Department of Radiation Oncology and Molecular Radiation Sciences, Johns Hopkins University School of Medicine, Baltimore, MD 21231; Department of Radiation Oncology and Molecular Radiation Sciences, Johns Hopkins University School of Medicine, Baltimore, MD 21231;; Department of Oncology, Johns Hopkins University School of Medicine, Baltimore, MD 21231;; Department of Mechanical Engineering, Whiting School of Engineering, Johns Hopkins University, Baltimore, MD 21218;; Department of Materials Science and Engineering, Whiting School of Engineering, Johns Hopkins University, Baltimore, MD 21218; Department of Mechanical Engineering, School of Science, Engineering, and Technology, The Pennsylvania State University—Harrisburg, Middletown, PA 17057

**Keywords:** spatially confined magnetic particle hyperthermia (SC-MPH), verification and validation, relay feedback, proportional–integral–derivative (PID) controller, magnetic nanoparticles (MNPs)

## Abstract

We have previously verified the capabilities of a prototype magnetic nanoparticle (MNP) heater (called HYPER) that can perform spatially confined heating; however, the design lacked temperature control capabilities. In this work, we designed, verified, and validated a relay-based autotuning proportional–integral–derivative (PID) controller to be used with the HYPER during in vivo experiments. The PID controller is an autotuning, relay-based controller with several design constraints: The controller must: (1) maintain tumor temperature within hyperthermic range of 41–46 °C; (2) rise time ≤ 5 min; (3) steady-state temperature must be within ±0.5° C of the setpoint; (4) standard deviation of steady-state temperature within ±0.5 °C; and (5) temperature overshoot within 5%. The relay-based autotuning PID controller was designed in LabVIEW^®^ with real-time thermal dose monitoring. Verification experiments were performed by heating aqueous suspensions of high-performance iron oxide MNPs. For validation, we injected the MNPs into tumor-bearing mice and analyzed the ability of the controller to maintain in vivo temperature. The results of the study show that controller was able to maintain the temperature within the hyperthermic range with a rise time ~4 min and steady-state error ~0.1 °C. Validation was performed on six mice, where four mice showed the temperature was maintained within design criteria and two mice partially met the design criteria. The autotuning controller can maintain the temperature within the design criteria and monitor thermal dose in real-time.

## 1 Introduction

Magnetic particle hyperthermia (MPH) is a minimally invasive therapy used to treat deep-seated tumors [1]. In MPH, magnetic nanoparticles (MNPs) are either directly or systemically delivered to the tumor site. An alternating magnetic field (AMF), typically in the radio frequency range of 100–350 kHz, is then applied [1,2]. When subjected to an AMF, MNPs exhibit a phase lag in their magnetic moment response due to Néel and Brownian relaxation mechanisms. This lag gives rise to a magnetic hysteresis loop, through which energy is dissipated in the form of heat, leading to a localized temperature increase at the tumor site [3–8].

However, MPH faces limitations due to the off-target distribution of MNPs [9,10]. Directly injected MNPs can diffuse into surrounding healthy tissue, while systemically administered MNPs tend to accumulate in the liver [11]. As a result, the application of AMF leads to heating not only within the tumor but also in healthy tissue and other organs where the particles have diffused. This off-target heating limits the clinical utility of MPH. This limitation can be addressed by spatially confining the heating zones. One method involves placing the MNPs between two opposing static magnets that generate magnetic fields stronger than the saturation magnetization of the MNPs. The interaction of these magnets creates a region with opposing magnetic fields, resulting in a volume with approximately zero magnetic field referred to as the field-free region (FFR). Within the FFR, magnetic moments are free to rotate in response to the AMF, enabling localized heating. Outside the FFR, the static magnetic field dominates, aligning the magnetic moments and minimizing their response to the AMF. This configuration enables selective heating within the FFR while reducing off-target heating in surrounding tissue known as spatially confined magnetic particle hyperthermia (SC-MPH) [12–15]. SC-MPH has been previously demonstrated in small animal models [16,17].

A prototype system is available that can demonstrate SC-MPH to a user-defined region of interest (ROI) [16]. The ROI is selected using two perpendicularly mounted cameras, and the gradient field strength is entered to define the volume to be targeted. Based on this input, the static magnets and patient bed reposition to create an FFR at the desired location. When an AMF is applied, heating occurs primarily within the ROI, with minimal heating in surrounding tissue. Despite this spatial targeting, the heating/temperature within the ROI is not actively controlled. Without temperature regulation, excessive heating may occur, potentially damaging nearby healthy tissue. Maintaining precise temperature control is important to maximize the tumor damage while minimizing harm to surrounding tissue, making reliable, user-friendly control systems essential for clinical translation.

Controllers have previously been explored for preclinical MPH. A proportional–integral–derivative (PID) controller, developed in alignment with Food and Drug Administration Design Control Guidance (which outlines regulatory best practices for medical device development), has been verified and validated [18,19]. In addition, other thermal therapy modalities, such as laser interstitial therapy and focused ultrasound, have employed temperature feedback PID and state feedback controllers in preclinical studies [20–28]. However, these controllers require manual input from a trained controls engineer during operation. As a result, translating such controllers into clinical settings presents challenges, particularly due to the need for specialized expertise.

The PID controller operates based on a user-defined temperature setpoint [29,30]. The controller calculates the error as the difference between the current temperature and the setpoint and determines the controller output as the sum of proportional, integral, and derivative actions. These actions are computed as follows: the proportional term is the product of the proportional gain and the current error; the integral term is the product of the integral gain and the accumulated error over time; and the derivative term is the product of the derivative gain and the rate of change of error.

Traditional methods for estimating PID gains (such as open-loop Ziegler–Nichols, Cohen–Coon) rely on the open-loop response of the system [31]. However, applying these methods in thermal therapy may result in overheating, which can damage healthy tissue. To overcome this challenge, feedback-based autotuning methods, such as relay autotuning, which switch the controller output fully ON or OFF based on temperature thresholds, offer a safer and more practical alternative [31,32]. In relay autotuning, a bang–bang control approach is used to maintain the temperature within a defined range. The resulting system response is then used to estimate appropriate PID gains. This method improves usability and reduces the need for a trained control engineer during treatment.

The objective of this study was to develop, verify, and validate an autotuning PID controller for SC-MPH. To achieve this, we implemented a relay-based autotuning PID controller using a combined NI LabVIEW^®^ to control the temperature by adjusting the AMF and python interface to select the ROI and reposition the static magnets [33].

## 2 Materials and Methods

### 2.1 Proportional–Integral–Derivative Controller Design and Construction

#### 2.1.1 Design Requirements.

The temperature feedback controller for MPH needs design specifications that address general hardware and software compatibility, as well as safety requirements, as outlined below. These specifications were based on the constraints of MPH and prior design of PID controller by Sharma et al. [18]:

- Capability to maintain temperature at a user defined target set point temperature ($T_{\text{sp}}$) within hyperthermic limits of 41–46 °C at a single sensor location.
- Rise time ($t_{r}$) to target temperature ($T_{\text{sp}}$) < 5 min.
- Overshoot $\left(M_{p}\right)\leq 5\%$.
- Steady-state temperature ($T_{\text{ss}}$) must be within ±0.5 °C of the temperature setpoint ($T_{\text{sp}}$).
- Standard deviation of the steady-state temperature ($T_{\text{ss},\sigma }$) must be within ±0.5 °C of the steady-state temperature ($T_{\text{ss}}$).

These requirements were selected based on clinical limits for hyperthermia. Temperatures above 46 °C typically fall under ablative therapy rather than hyperthermia. In addition, most treatments are limited to approximately 30 min to minimize the duration of anesthesia. A rise time exceeding 5 min would reduce the effective treatment period. Overshoot is also constrained to minimize damage to surrounding healthy tissue.

#### 2.1.2 Safety Requirements.

Additionally, we implemented safety requirements to limit unintended temperature deviations within the tumor and surrounding tissue and to protect the system hardware from potential damage.

- A user-defined maximum temperature within the treatment region to limit power and prevent high heating at the feedback sensor.
- A rate limiter of 5mT/s on the amplitude of AMF to prevent fluctuations from higher-order harmonics. The derivation for this 5mT/s rate limiter has been previously demonstrated [18].

#### 2.1.3 Design of Autotuning Proportional–Integral–Derivative Controller.

A PID controller was designed to control the temperature within the design criteria [18]. The governing equation for the PID controller is shown in the following equation:

(1) $$U_{\text{ctrl}}=K_{c}\cdot \left(e(t)+\frac{1}{T_{i}}\cdot \int_{0}^{t}e(\tau )d\tau +T_{d}\cdot \frac{d\;e(t)}{dt}\right)$$

where $K_{c}\left(\text{V}\cdot (\text{K}{)}^{-1}\right)$ is the proportional gain, $e(t)(\text{K})=T_{\text{SP}}-T_{P_{i}}$ is the error (i.e., the difference between the temperature setpoint $T_{SP}$ and the control temperature of the ith sensor) $T_{P_{i}},T_{i}(\text{s})$ is the integral time constant, and $T_{d}(\text{s})$ is the derivative time constant.

**Fig. 1:**
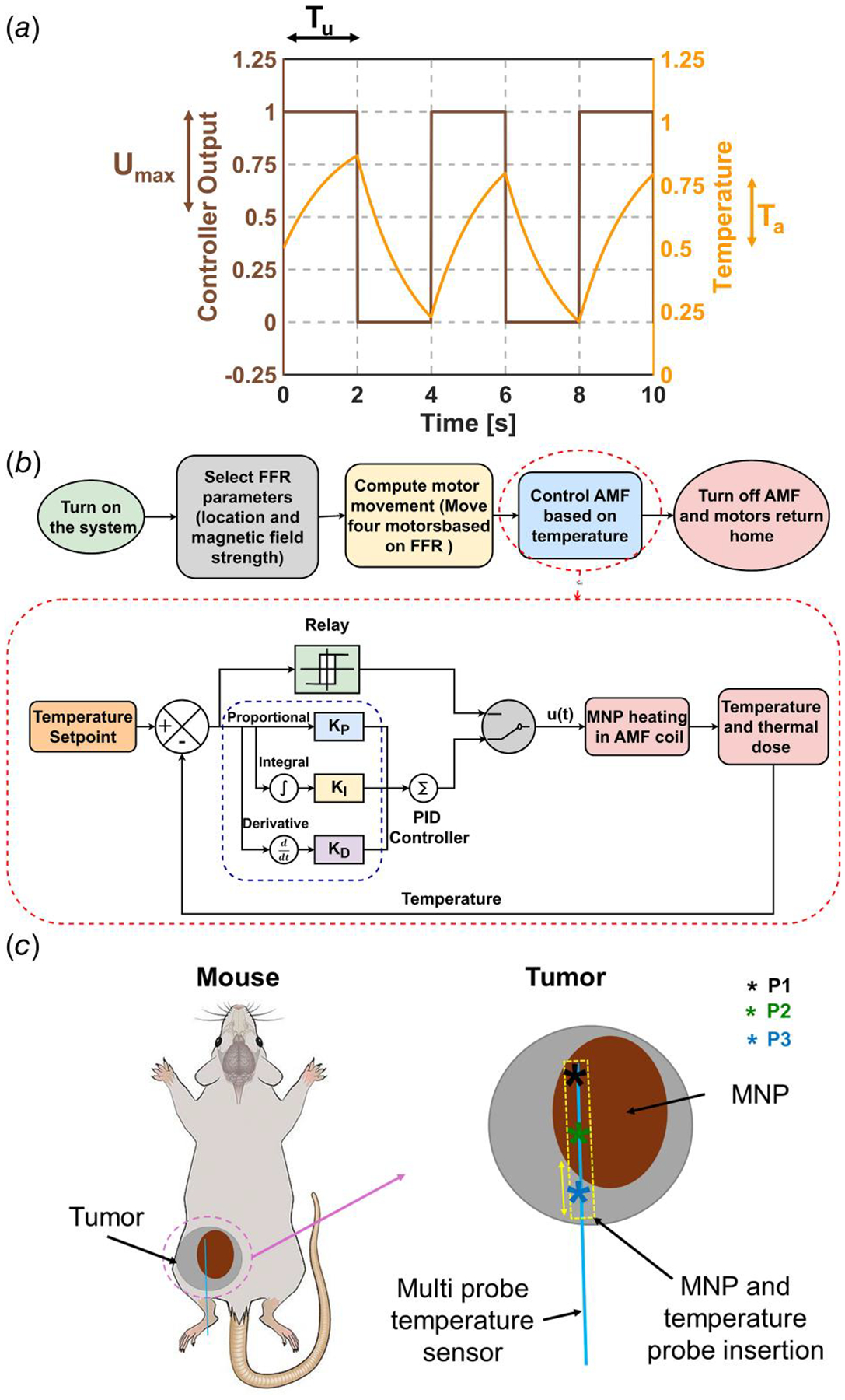
Setup used for design of PID controller and in vivo validation experiments. (*a*) Determination of ultimate period ($T_{u}$) and ultimate gain ($K_{u}$) using maximum magnetic field ($U_{\text{max}}$) and amplitude of temperature ($T_{a}$) using a relay based controller. (*b*) Implementation of an autotuning PID controller in LabVIEW^®^ to regulate the AMF based on temperature feedback. The process starts with selecting the FFR from a camera image, followed by precise motor positioning through a python interface. Once positioned, the relay-based autotuning method determines the optimal PID gains by analyzing the system’s oscillatory response. These gains are then used by the PID controller to compute the control output as a sum of proportional, integral, and derivative terms. This closed-loop system ensures precise temperature regulation for SC-MPH. (*c*) Schematic (for representative purposes) of the experimental setup used to validate the autotuning PID controller for SC-MPH. MNPs are injected using a syringe pump, and a similar path is used to insert the temperature sensor into the tumor. Temperature control is based on feedback from the sensor that records the fastest rise in temperature.

To obtain PID control gains, a relay-based autotuning method was used [31]. In relay-based autotuning, a controller signal is provided to maintain the temperature within the specified limit using a bang–bang controller as shown in Fig. 1(a). The temperature response is used to obtain the ultimate period and gain which are used to obtain the PID gains. The evaluation of ultimate period $T_{u}$ was obtained from the relay-based response, and the determination of ultimate gain is shown in the following equation:

(2) $$K_{u}=\frac{4\cdot U_{\text{max}}}{T_{a}\cdot \pi }$$

where $K_{u}$ is the ultimate gain, $U_{\text{max}}$ is the maximum controller output, and $T_{a}$ is the amplitude of deviation of the temperature from the temperature setpoint during autotuning.

The ultimate period and the gain were used to obtain the proportional gain, integral time constant, and derivative time constant as shown in Eqs. (3)–(5) using modified closed-loop Ziegler–Nichols methods for system with moderate rise time to conservatively reach to the setpoint with emphasis on patient safety [31,32]

(3) $$K_{c}=0.25\cdot K_{u}$$

(4) $$T_{i}=0.5\cdot T_{u}$$

(5) $$T_{d}=0.12\cdot T_{u}$$

#### 2.1.4 Integration of Real-Time Thermal Dose Calculation.

Thermal dose (CEM43) in MPH is calculated as the cumulative exposure time at a specific temperature integrated over time. The thermal dose is determined using the following equation [34]:

(6) $$\text{CEM43}=\int_{t=0}^{t=t_{f}}B^{\frac{\left(43\left({}^{\circ}\text{C}\right)-T(x,y,z,t)\right)}{1\left({}^{\circ}\text{C}\right)}}dt$$

The constant B is defined as 0.5 for $T>43^{\circ }\text{C}$ and 0.25 for $T\leq 43^{\circ }\text{C}$. Since temperature is measured at discrete time steps, the thermal dose is numerically approximated using the following equation:

(7) $${\text{CEM}43}_{T}=\sum_{i=0}^{t}\left[B^{\frac{\left(43\left({}^{\circ}\text{C}\right)-T_{i}(x,y,z,t)\left({}^{\circ}\text{C}\right)\right)}{1\left({}^{\circ}\text{C}\right)}}\right]\times \Delta t$$

where $\text{CEM}43_{T}$ at time t is summation of the product of exponential of temperature at discrete times $T_{i}(x,y,z,t)$ with sampling frequency Δt(Δt~0) at the specific location.

#### 2.1.5 Hardware: HYPER.

We provided a full description of the HYPER prototype in our previous study [16]. Briefly, HYPER allows the user to select the location and size of the FFR for SC-MPH. A set of motors ensures the user selected location coincides with the center of the FFR, as well as adjusts the FFR volume by varying the distance between the two permanent magnets. After setting the sample location and FFR size, the MNPs within the FFR are heated. Previously, the temperature measurement was limited to two independent sensors, but for the current experiments, the sensor has been upgraded. We installed a multipoint temperature sensor (FISO EVO^®^ THR-MULTI-8, FISO, Quebec, Canada), capable of measuring temperature at eight points spaced 3 mm apart [35]. The temperature signals from the fiber optic sensor were conditioned using the data acquisition (FISO Evo SD-4 chassis, FISO, QC, Canada), which converted the signals into a 0–5 V analog voltage output, which linearly scaled between 10 °C and 90 °C [35]. The sensor was interfaced with the data acquisition system. Due to current limitations in the data acquisition system, only three measurement points were utilized; however, the system allows the user to select points of interest based on experimental requirements.

#### 2.1.6 Software.

The software for the device was divided into two domains: (i) FFR adjustment and (ii) temperature feedback control.

- FFR adjustment
  The sample holder was positioned at the desired location using the existing python-based code, as illustrated in our previous study [16]; however, we omitted the portion of the code that controlled the RF amplifier. In this case, the user inputs the coordinates and gradient field value, and the system adjusts the sample position and FFR volume accordingly and holds those values until the code is aborted.
- Temperature feedback control
  Temperature control was implemented using a LabVIEW^®^ interface (National Instruments, Austin, TX). The user specifies the temperature setpoint, safety temperature, output range, initial PID gains, temperature sensor offsets, and selected the sensor for control. System activation was initiated by enabling the tuning mode in the interface, triggering relay-based autotuning to the setpoint temperature. This process involved applying a step response at the maximum output range, followed by AMF modulation to determine appropriate PID gains. The sampling frequency was set to 10 Hz, as established in previous work by Sharma et al. [18]. Once tuning was completed, the LabVIEW^®^ user interface indicator signaled the user to disable tuning, after which the determined PID gains were applied. The system automatically shuts down if the temperature exceeds the safety threshold. Temperature data, cumulative equivalent minutes at 43 °C (CEM43) for all three sensors, and controller output were recorded in a text file for further analysis. A diagram of PID controller can be seen in Fig. 1(b).

### 2.2 Controller Verification and Validation

#### 2.2.1 Magnetic Nanoparticles Used.

For the verification and validation experiments, we used Synomag^®^ nanoparticles (micromod Partikeltechnologie, GmbH, Rostock, Germany). The verification experiments used Synomag^®^-COOH (50 nm; Lot#: 12622103-01), as received, at a reported concentration of $80\;{\text{mg}}_{\text{Fe}}/\text{mL}$. For the validation experiments, we used Synomag^®^-S (90 nm; Lot#: 43623105-01) at a concentration of $70\;{\text{mg}}_{\text{Fe}}/\text{mL}$, which was verified using ferene-s assay before intratumor injection [36].

#### 2.2.2 Controller Verification.

Magnetic nanoparticles at $80{\text{mg}}_{\text{Fe}}/\text{mL}$ concentration in a 2 mL Eppendorf tube were exposed to an AMF to regulate temperature. Among the three available sensors to acquire data on the temperature sensor, we used the first, which was fully immersed in the nanoparticle sample. The autotuning PID controller was activated, and temperature was set at 35, 40, and 45 °C. To assess the adaptability of the autotuned gains, the setpoint was varied, and the steady-state error along with the standard deviation of the results was recorded. This provided a quantitative comparison of the impact of setpoint changes. Additionally, the gains were retuned at a higher setpoint, and the results were compared to those obtained without altering the PID gains.

#### 2.2.3 Mouse Model for in vivo Controller Validation.

The study procedures for this work were performed in accordance with the Johns Hopkins University Institutional Animal Care and Use Committee (IACUC#: MO24M16). For this work, we used 9, 8-week-old female BALB/c mice (Jackson Laboratory, Bar Harbor, ME). We monitored the mice daily for signs of pain or distress, maintained a 12 h light/12 h dark cycle, and provided food and water ad libitum. We procured a vial of 4T1 murine mammary carcinoma cells (ER/PR/HER2 negative) from the American Type Culture Collection (ATCC, Manassas, VA). We cultured the cells according to the supplier recommendations: Roswell Park Memorial Institute 1640 media with 10% fetal bovine serum. Each mouse was then injected with 50,000 cells in 50 *μ*L phosphate buffered saline subcutaneously into the right thigh. We measured the tumors until they reached at least 200 mm^3^ in size, but not greater than 700 mm^3^.

#### 2.2.4 Intratumor Magnetic Nanoparticle Injections.

We anesthetized the mice with 1–2% isoflurane mixed with ${\text{O}}_{2}$, which we delivered through a nose cone. While anesthetized, we injected high concentration ($70\;{\text{mg}}_{\text{Fe}}/\text{mL}$) MNPs intratumorally at a rate of 1μL/min using a syringe pump (Pump 11 Elite, Harvard Apparatus, Holliston, MA). After injection, we waited for an additional 15 min before removing the needle to allow the particles to disperse throughout the tumor.

#### 2.2.5 Controller Validation Experiments.

Immediately after MNP injection, the mice were warmed until their core body temperature was between 37 and 38 °C. After warming, each anesthetized mouse was placed on a heated sample holder, which also contained a nose cone to provide constant anesthesia. While anesthetized, a fiber optic rectal sensor was inserted. Additionally, we inserted the FISO multisensor probe manually intratumorally along the length of the tumor, parallel to the body along the same path of MNPs injection as shown in Fig. 1(c). Hence, the sensor position in each animal varied depending upon the position of injection of MNP and operator skill. Once the sensor was secured, the mouse was placed within the HYPER, with the FFR centered on the approximate geometric tumor center. Inside the HYPER, 37 °C water circulated within the RF coil to provide a warm environment necessary to prevent the core body temperature from falling. Additionally, we monitored the core body temperature to not exceed 39 °C throughout the test.

When the rectal and intratumor temperatures stabilized after 5–10 min, we selected the PID output range and enabled the autotuner, which began to heat the mouse and converge on the optimal PID gains. Upon completion of the autotuning stage, we turned it OFF and allowed the system to converge on the given setpoint (45 °C) until a thermal dose of CEM43 = 30 min was achieved. When the thermal dose was reached, the heating was then stopped, and the mouse was removed from the HYPER.

#### 2.2.6 Statistical Analysis.

Temperature data were compiled and saved as a .txt file from the LabVIEW^®^ interface. We imported the saved data into matlab and used custom code to plot it and extract our design-specification metrics. We calculated rise time ($t_{r}$) as the interval during autotuning for the temperature to go from 10% to 90% of the difference between the initial value and the setpoint. We determined percentage overshoot from the peak temperature reached during treatment, steady-state temperature ($T_{\text{ss}}$) and deviation ($T_{\text{ss},\sigma }$) from the temperature recorded after tuning was complete. The obtained data were analyzed for normality using Ryan–Joiner (RJ) test for normality using minitab, and 95% confidence intervals for verification experiments were reported.

## 3 Results

### 3.1 Controller Verification.

**Fig. 2:**
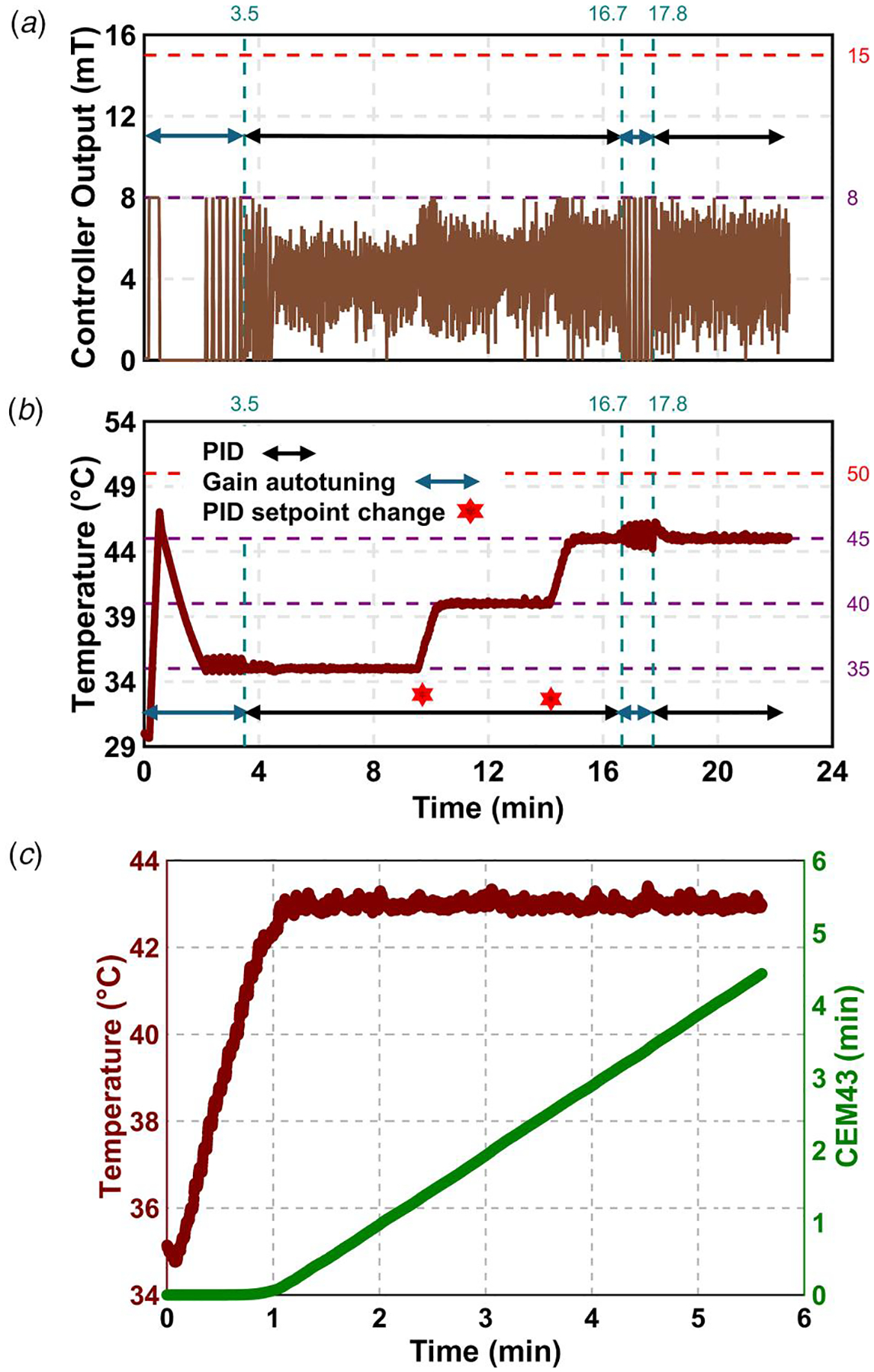
Verification of the autotuning PID controller at multiple setpoints and thermal dose (CEM43) calculator at 43 °C using a sample of high concentration of MNPs (n=3). (*a*) Amplitude of AMF: Initially, the AMF amplitude was turned ON and OFF to determine the PID gains using relay autotuning method. Next, the controller AMF amplitude was calculated based upon the temperature feedback using PID controller at multiple temperature setpoints of 35, 40, and 45 °C. Autotuning was reinitialized again at 45 °C where the gains were obtained again. (*b*) Temperature: Initially, the setpoint was set at 35 °C, and the autotuning process was initiated, causing fluctuations as the controller turned ON and OFF. The relay feedback determined the controller gains (~3.5 min), and a green signal on the user interface confirmed successful tuning. The tuned gains maintained 35 °C for ~5 min. The same gains were applied to setpoints of 40 °C and 45 °C, maintaining the setpoint for ~5 min and 2 min, respectively. Subsequently, the autotuning PID was turned ON at 45 °C, obtaining new gains more quickly (~1.5 min), showing the advantage of a good initial guess in optimizing gain determination. (*c*) The above temperature verification experiment confirmed that the gains obtained for one setpoint could be applied to multiple setpoints (e.g., gains from 35 °C were successfully used for 40 °C and 45 °C). The previously tuned gains maintained the temperature at 43 °C. Since CEM43 is an exponential function of temperature, its initial rate of change was ~0 (CEM43/min). Once CEM43 reached the setpoint, the slope was constant(~1(CEM43/min)).

We verified the autotuning PID controller using probe P1, as shown in Figs. 2(a) and 2(b) and Fig. S1 available in the Supplemental Materials on the ASME Digital Collection for three iterations. Initially, we set the controller output range to 0–15mT. However, due to the 5mT/s rate limit, this caused the temperature to rise rapidly. To resolve this, we adjusted the output range through trial and error and limited it between 5 and 8 mT. We set the initial temperature setpoint at 35 °C and used the relay autotuning method to obtain the PID gains. The system was maintained at 35 °C for 5 min.

To assess controller performance, we increased the setpoint to 40 °C while keeping the same gains. The system reached the new setpoint and held it for 5 min. We then raised the setpoint to 45 °C and maintained it for 2 min with the same gains. To improve performance at this higher setpoint, we reinitiated the autotuning process and updated the PID gains. With the new gains, the system stabilized at 45 °C and held the temperature for 5 min.

Table 1 summarizes the performance metrics for all three setpoints. The results indicate that a single set of PID gains can effectively control the system due to its linear behavior. In all cases, the standard deviation of the temperature remained below the sensor’s measurement uncertainty of 0.2 °C.

Next, we plotted probability distribution fitted against normal distribution as shown in Fig. S2 available in the Supplemental Materials and reported RJ coefficient in Table S1 available in the Supplemental Materials. The results suggest that the distribution of temperature around the setpoint is not normally distributed as the peak had higher probability showing the effectiveness of controller and temperatures two standard deviations higher than setpoint were more frequent than two standard deviations lower than the setpoint as rate of rise of temperature was much greater than the rate of decrease of temperature. We have reported 95% confidence intervals in Table 1. Additionally, we were within the uncertainty of temperature sensor. Hence, further statistical analysis had limited interpretability due to distributional violations and sensor uncertainty.

To verify CEM43 monitoring, a setpoint temperature of 43 °C was used. The controller reached a steady-state temperature of 43 °C in ~65 s, with a steady-state error of 0.003 °C and a standard deviation of 0.097 °C, as shown in Fig. 2(c). This temperature was maintained for ~270 s. Using Eq. (7), the CEM43 calculator in LabVIEW determined the experimental thermal dose from 65 to 335s to be 4.31min. When calculated analytically using Eq. (6), where a constant temperature of 43 °C is assumed over the same time period, the CEM43 value was determined to be 4.41 min. The resulting 2.3% relative error between our experimentally calculated and the analytically calculated CEM43 value verified the functionality of our integrated CEM43 monitor.

### 3.2 Controller Validation.

**Fig. 3:**
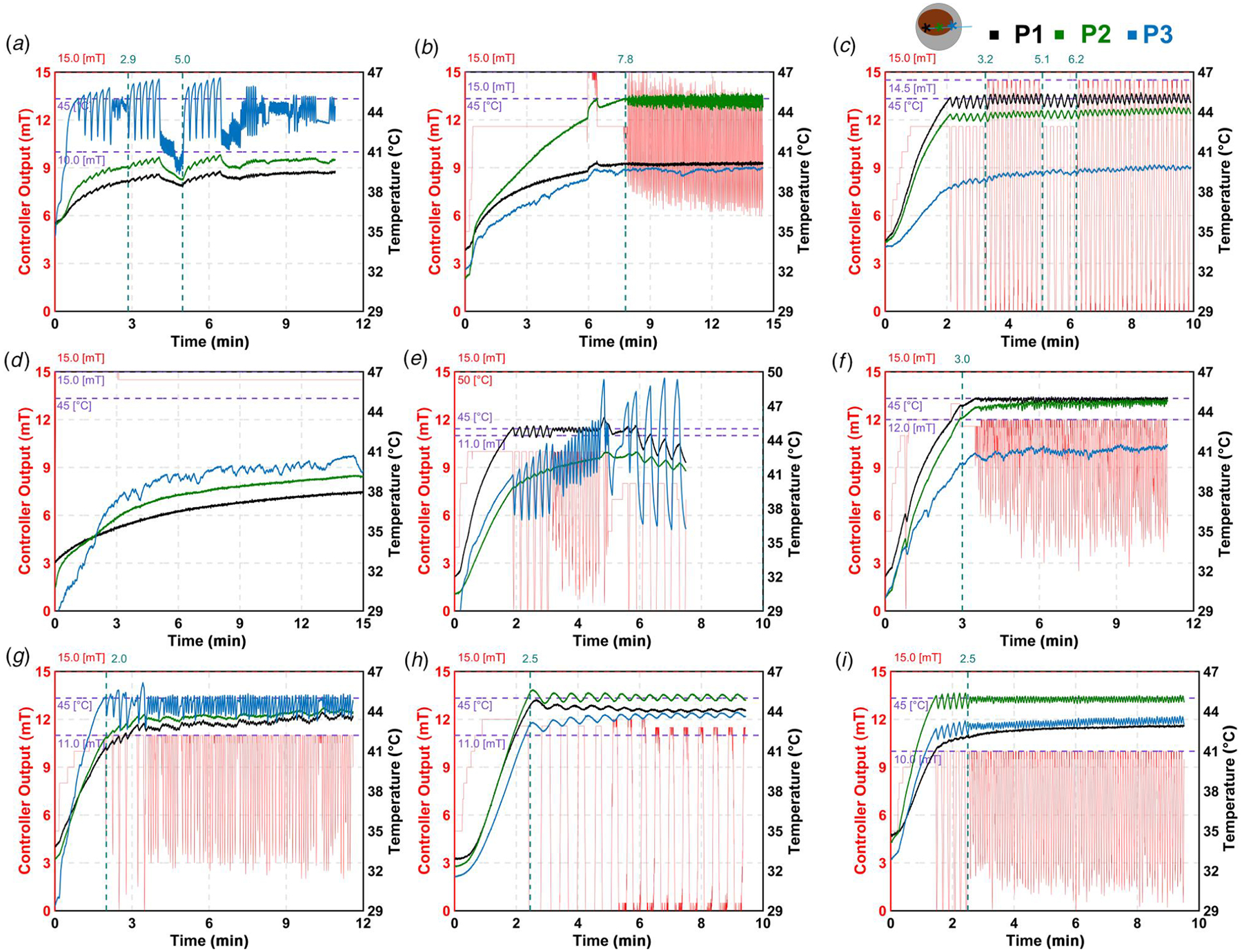
Validation of the PID control in murine breast cancer model (n=9), with various temperature control sensors (P1, P2, and P3). The temperature was controlled based on the sensor that recorded the fastest temperature rise. (*a*) Mouse 1; (*b*) Mouse 2; (*c*) Mouse 3; (*d*) Mouse 4; (*e*) Mouse 5; (*f*) Mouse 6; (*g*) Mouse 7; (*h*) Mouse 8; and (*i*) Mouse 9.

Figure 3 shows the chronological heating data for each mouse included in this study. Table 2 summarizes the controller parameters and performance for the mice shown in Fig. 3. Four of the heated mice met all design specifications shown in Fig. 3: mouse 3 (Fig. 3(c)), mouse 6 (Fig. 3(f)), mouse 8 (Fig. 3(h)), and mouse 9 (Fig. 3(i)), and two mice, mouse 2 (Fig. 3(b)) and mouse 7 (Fig. 3(g)), partially met the criteria. Three of the mice resulted in poor control response for a variety of reasons as explained below are shown in Fig. 3: mouse 1 (Fig. 3(a)), mouse 4 (Fig. 3(d)) and mouse 5 (Fig. 3(e)). Thermal dose plots for all nine mice are shown in Fig. S3 available in the Supplemental Materials.

Initially, the controller output range was set conservatively, as high temperature slopes were observed during verification experiments. To address this, the maximum controller output gradually increased in steps, as shown in Fig. 3(c). However, in some cases, the temperature showed undamped oscillations, resulting in an oscillating response (Fig. 3(c)). Therefore, we tuned the system again using autotuning as shown in Fig. 3(c) for ~5 min but still the oscillations did not dampen. Hence, we did not use autotune feature two times in Fig. 3(c).

In Fig. 3(g), we noted that the temperature response remained undamped over time. This could be due to the backflow of MNPs leading to localized increase in the MNP concentration which leads to increased heat source in this region. Similar behavior was observed in other mice as well (Fig. 3(a)), indicating that temperature control away from the tumor center could be more challenging.

Figure 3(e) shows a mouse in which the temperature at sensor 3 rose rapidly, prompting a switch in the control sensor during treatment. Following this switch, the amplitude of temperature oscillations increased over time. As a result, the treatment was terminated after reaching a thermal dose of 30 min. In another case (Fig. 3(d)), the tumor did not heat as expected, and the temperature remained below 40 °C. This was likely due to the injected MNPs diffusing out of the tumor, rather than remaining within the target region.

## 4 Discussion

Spatially confined magnetic particle hyperthermia has been demonstrated previously but lacks feedback temperature control. Hence, in this study, we addressed this gap by designing, verifying, and validating an autotuning relay feedback PID controller for temperature regulation. Verification experiments showed that the PID controller achieved the target temperature within ~4 min(≤5 min) and maintained the temperature within 0.1 °C of the setpoint (≤0.5 °C).

These results demonstrate accurate and fast temperature regulation; however, during the tuning phase, a large overshoot was observed. The overshoot was due to the combined effects of using a high-concentration MNP sample and a built-in rate limiter for the RF power amplifier. Since we used the MNPs at stock concentration for verification ($80\;{\text{mg}}_{\text{Fe}}/\text{mL}$), this led to an exorbitantly high temperature change during heating. The rate limiter, set at 5 mT/s, was included to prevent hardware damage, but introduced a delay when turning OFF the heating completely before the temperature exceeded 5% of temperature setpoint. In clinical treatments, the target intratumor MNP concentration is much lower, at around $15-20\;{\text{mg}}_{\text{Fe}}/\text{mL}$; this is due to intraparticle diffusion within the tumor, nanoparticle extravasation, and leakage from the injection site [9].

At these lower in vivo concentrations, the reduced heating power may increase rise time, as a lower heat source requires more time to achieve the target CEM43 at a sensor location (Tables 1 and 2). This suggests that the system should operate safely with <5% overshoot under standard conditions, even with the limiter, as shown with the validation experiments. Nevertheless, this distributed heating profile could be advantageous, as diffused particles are more likely to produce a uniform volumetric CEM43. Importantly, a relay autotuned PID controller, which is particularly effective for slower systems, should still regulate temperature within the defined steady-state error limits.

The use of the same PID gains across different setpoints during verification indicates the system behaves approximately linearly. Although the rate limiter on amplitude of AMF introduces nonlinearity, a second-order approximation still holds which we have demonstrated in our previous study to some extent [18]. This suggests that tuning can be performed at a lower setpoint, and the same gains can be applied at higher setpoints increasing the safety of SC-MPH.

Validation was performed on nine mice with varying tumor sizes. Four met the performance criteria, two partially met them: one with a rise time above 5 min, and one with a steady-state deviation above 0.5 °C, two mice showed uncontrolled heating, and one mouse did not heat above ~41 °C. Control using sensors 1 and 2 consistently met the criteria. In contrast, control using sensor 3 failed in all cases. One possible reason is the backflow of MNPs. Intratumoral injections of MNPs can lead to backflow during treatment, and sensor 3 is often positioned near this region. This may cause a local increase in MNP concentration, leading to uncontrolled heating.

In one case (Fig. 3(e)), the user switched control from sensor 1 to sensor 3 after the latter registered a higher temperature. However, since the controller had been tuned based on sensor 1, switching sensors without resetting the integral term caused poor regulation of temperature. This highlights a key limitation: changing the control sensor during treatment is not recommended. If unexpected temperature rises occur at noncontrolling sensors, it is preferable to halt treatment, as was necessary in this case.

The robustness of the autotuning PID controller also depends on the number of relay oscillations during tuning. High-concentration samples produce higher number of oscillations and more reliable gain estimates but risk overshoot. In contrast, low-concentration (slow-heating) samples produce fewer or no oscillations, resulting in less reliable gains. Therefore, autotuning is most effective at intermediate MNP concentrations ($5-20\;{\text{mg}}_{\text{Fe}}/\text{mL}$), which are also preclinically relevant [10]. Moreover, low-heating samples may not require tuning, as default PID gains may maintain the desired temperature.

Ziegler–Nichols and Åström–Hägglund approaches typically result in high proportional gains, which make the controller more aggressive and prone to overshoot during heating [29–31]. Hence, we used a modified Ziegler–Nichols relay feedback autotuning strategy based on the closed-loop tuning method. This is a conservative tuning strategy to limit overshoot and reduce the risk of thermal damage to sensitive structures such as blood vessels. This conservative approach reduced the proportional gain ($K_{c}$), resulting in an overdamped system response (Tables 1 and 2). While this can lead to a longer rise time, only one mouse in the study had a rise time exceeding 5 min, indicating that the system remained within design specifications in most cases. Overdamped behavior was observed in five of six cases (Table 1). Although this may increase treatment duration, as thermal dose accumulates exponentially with temperature (Eq. (6)), the deviations remained within the precision limits of the temperature sensors and are acceptable for MPH. Additionally, the reduced derivative gain minimized oscillations and improved the system’s robustness by enhancing its ability to reject disturbances.

Minimizing user input is important for clinical adoption. The current controller requires the user to input a power range. Future iterations could automate this step by analyzing the early heating response from the hottest sensor. By setting a maximum rate of temperature rise and assuming approximate linearity, the system could estimate the power range and prevent overshoot without user intervention.

In this study, validation was performed without spatial MNP distribution data. However, MNPs distribute nonhomogenously within tumors depending on the fate of local tissue properties and MNP injection parameters, making spatial information vital for accurate temperature prediction and control [9,10]. Magnetic particle imaging (MPI) can provide this spatial mapping and has been used to generate individualized heating profiles [37,38]. This supports the development of temperature-controlled treatment planning, a necessary step towards patient-specific therapy.

Patient-specific implementation requires accurate models of heat transfer, typically developed using finite element analysis (FEA) [10,39]. While effective, FEA is computationally expensive and impractical for real-time use. Reduced-order models (ROMs) are a potential alternative, but traditional ROMs lack physical accuracy. Physics-informed deep learning models offer a promising solution by combining physical laws with data-driven learning [40–42]. These models can produce fast, accurate predictions, enabling real-time adaptive planning.

Still, real-time treatment planning implementation also requires adaptive control. Sensor placement and tissue properties vary among patients and must be estimated during treatment. Applying a short heating pulse and observing the temperature response allows inverse parameter estimation to identify these properties [27,43–46]. With this information, treatment can be adjusted in real-time. However, adjustments must be limited, as perfusion effects are nonlinear and difficult to predict.

Currently, the control system relies on three temperature sensors placed 3 mm apart, which are manually inserted by the operator along the path of MNP injection. However, sensor positioning can vary between animals depending on tumor geometry and injection accessibility. Moreover, this study did not employ MPI guidance or stereotactic methods for sensor placement. Future work should incorporate stereotactic, MPI-guided robotic injection of both MNPs and sensors to enable more consistent placement and improve the effectiveness of treatment planning.

Despite these improvements in placement, a further limitation remains: the present SC-MPH framework uses only one temperature sensor for feedback, making it a single-input, single-output system, while the remaining sensors function only as passive monitors. A more advanced strategy would use all sensors and their temperature rates through state-space or predictive control. Techniques like model predictive control (MPC) can predict future temperatures and adjust heating accordingly, preventing potential temperature over-shoots [47,48]. Predictive strategies would enhance system reliability, particularly when combined with spatial data on MNP distribution.

The current system performs heating with static FFR, but tumors and MNP distributions are often nonuniform. Previous work has shown that steered heating within tumor can improve tumor coverage [49]. Future systems should allow dynamic steering of heating zones to maximize coverage while protecting healthy tissue.

Scaling this approach to humans introduces new challenges. In this study, rectal temperatures stayed within safe limits, indicating minimal eddy current heating. However, at larger scales, eddy current heating increases significantly due to body size [50]. Pulse width modulation has been proposed to control this effect [51,52], but selecting a patient-specific pulse width is complex. MPC could address this by predicting when heating should turn OFF or ON, reducing eddy current heating without user input [53].

For clinical translation, a patient-specific, MPC-based treatment plan is essential. This requires surrogate models to replace FEA and reduce computational demands. While real-time MPC remains challenging, offline predictive planning using patient-specific data offers a feasible path forward [54,55].

## 5 Conclusion

The verification and validation of the autotuning PID controller shows that temperature can be maintained within the designed criteria for SC-MPH. The controller successfully estimates the PID gains, enhancing the user experience and reducing the need for a trained controls engineer. However, estimating the initial controller range remains a challenge. Future work should focus on automating the estimation of the controller range, dynamically adjusting the FFR to maximize the coverage index, and implementing advanced control strategies such as MPC to further improve user experience and minimize the reliance on a controls engineer in preclinical settings.

## Supplementary Material

Supplementry Material

## Figures and Tables

**Table 1:** Controller gains obtained through autotuning and evaluation of design criteria for verification of controller

Figure 2			Iteration		
			1	2	3
$t_{r}$^a^ (min)			0.22	0.03	0.03
$T_{\text{SS}}\pm T_{\text{ss},\sigma }$ (°C)	35		35.00±0.04	35.07±0.06	35.06±0.07
	40		40.00±0.07	40.08±0.11	40.07±0.11
	45	A1	44.98±0.07	45.06±0.13	45.03±0.10
		A2	45.01±0.09	45.05±0.09	45.08±0.13
95% CI^a^ of	35		(34.92, 35.08)	(34.95, 35.19)	(34.92, 35.20)
$T_{\text{SS}}$ (°C)	40		(39.86, 40.14)	(39.86, 40.30)	(39.85, 40.29)
	45	A1	(44.84, 45.15)	(44.81, 45.31)	(44.83, 45.23)
		A2	(44.83, 45.19)	(44.87, 45.23)	(44.83, 45.33)
OS^a^ (%)			34.41	13.81	16.48

a $t_{r}$: rise time, $T_{\text{ss}}$: steady-state temperature, $T_{\text{ss},\sigma }$: standard deviation, OS: percentage overshoot, and CI: confidence interval.

**Table 2:** Controller gains obtained through autotuning and evaluation of design criteria for validation of controller

Figure 3 panel	a		b	c		d	e	f	g	h	i
	1	2		1	2						
$K_{c}$ (V/°C)	0.03	0.03	1.13	0.12	0.12	N/A	0.12	1.29	0.04	0.22	0.10
$T_{i}$ (min)	0.16	0.16	0.02	0.15	0.15	N/A	0.15	0.02	0.20	0.28	0.13
$T_{d}$ (min)	0.04	0.03	0.01	0.04	0.03	N/A	0.03	0.00	0.04	0.07	0.03
Control sensor	3		2	1		3	1, 3	1	3	2	2
Tumor volume (mm^3^)	283.25		293.90	279.83		248.60	525.80	612.50	350.80	643.90	470.00
$t_{r}$^a^ (min) (≤5.00 min)			5.96	1.77		N/A		2.66	1.53	2.13	1.23
$T_{\text{SS}}\pm T_{\text{ss},\sigma }$^a^ (°C) (44.50–45.50 °C)	N/A		44.82±0.32	44.99±0.22		N/A	N/A	44.92±0.09	44.47±0.50	45.06±0.18	44.90±0.14
OS^a^ (%) (≤5.00%)	N/A		0.96	1.01		N/A		0.29	2.58	1.41	0.86
Design specs met?	No, user learning curve		No, rise time ≥ 5.00 min	Yes		No, heating was not sufficient to reach setpoint temperature	No, user error	Yes	No, steady state deviation ≥ 0.50 °C	Yes	Yes

a $t_{r}$: rise time, $T_{\text{ss}}$: steady-state temperature, $T_{\text{ss},\sigma }$: standard deviation, and OS: percentage overshoot.

## Data Availability

The raw data supporting the conclusions of this article will be made available by the authors, without undue reservation.
