# Supplementary material for: Verification and Validation of an Autotuning Proportional–Integral–Derivative Controller for Spatially Confined Magnetic Particle Hyperthermia
Source: J Med Device. Author manuscript; Available in PMC 2026 Jan 31. (PMC12755161; doi:10.1115/1.4070364)
Supplement: Supplementry Material [file NIHMS2140034-supplement-Supplementry_Material.docx]

**Supplementary Information**


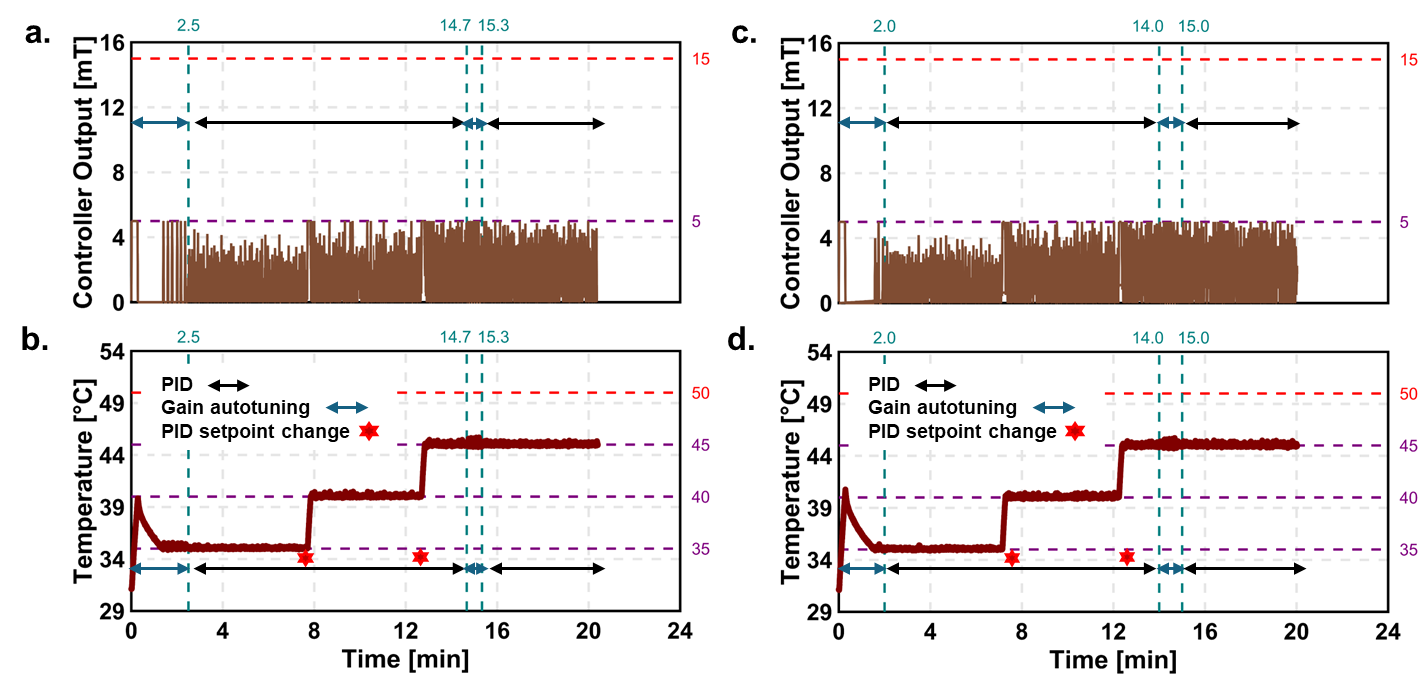


**Figure S1: Verification experiment:** Iteration 2 **a.** Controller output **b.** Temperature Iteration 3 **c.** Controller output **d.** Temperature


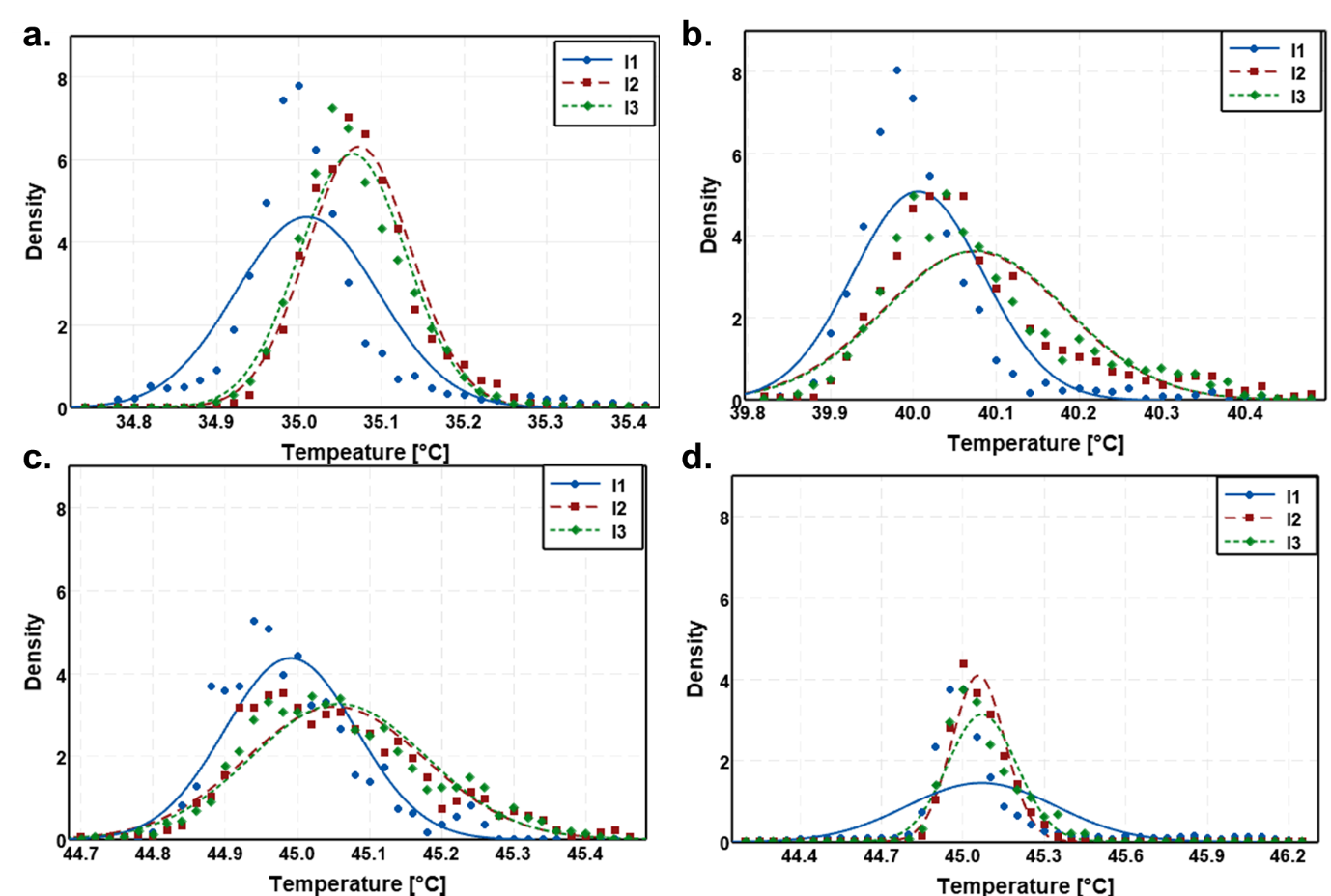


**Figure S2: Probability distribution of temperature at four setpoints during each iteration (I) for verification experiment.** **a.** 35 [°C] **b.** 40 [°C] **c.** 45 [°C] Attempt 1; **d.** 45 [°C] Attempt 2.

**Table S1:** Normality testing using RJ statistics at multiple setpoints for three iterations.

| Temperature Setpoint | 35 [°C] | 40 [°C] | 45 [°C] Attempt 1 | 45 [°C] Attempt 2 |
| --- | --- | --- | --- | --- |
| Iteration 1 | 0.95 | 0.92 | 0.98 | 0.88 |
| Iteration 2 | 0.99 | 0.96 | 0.99 | 0.99 |
| Iteration 3 | 0.99 | 0.97 | 0.99 | 0.98 |

**
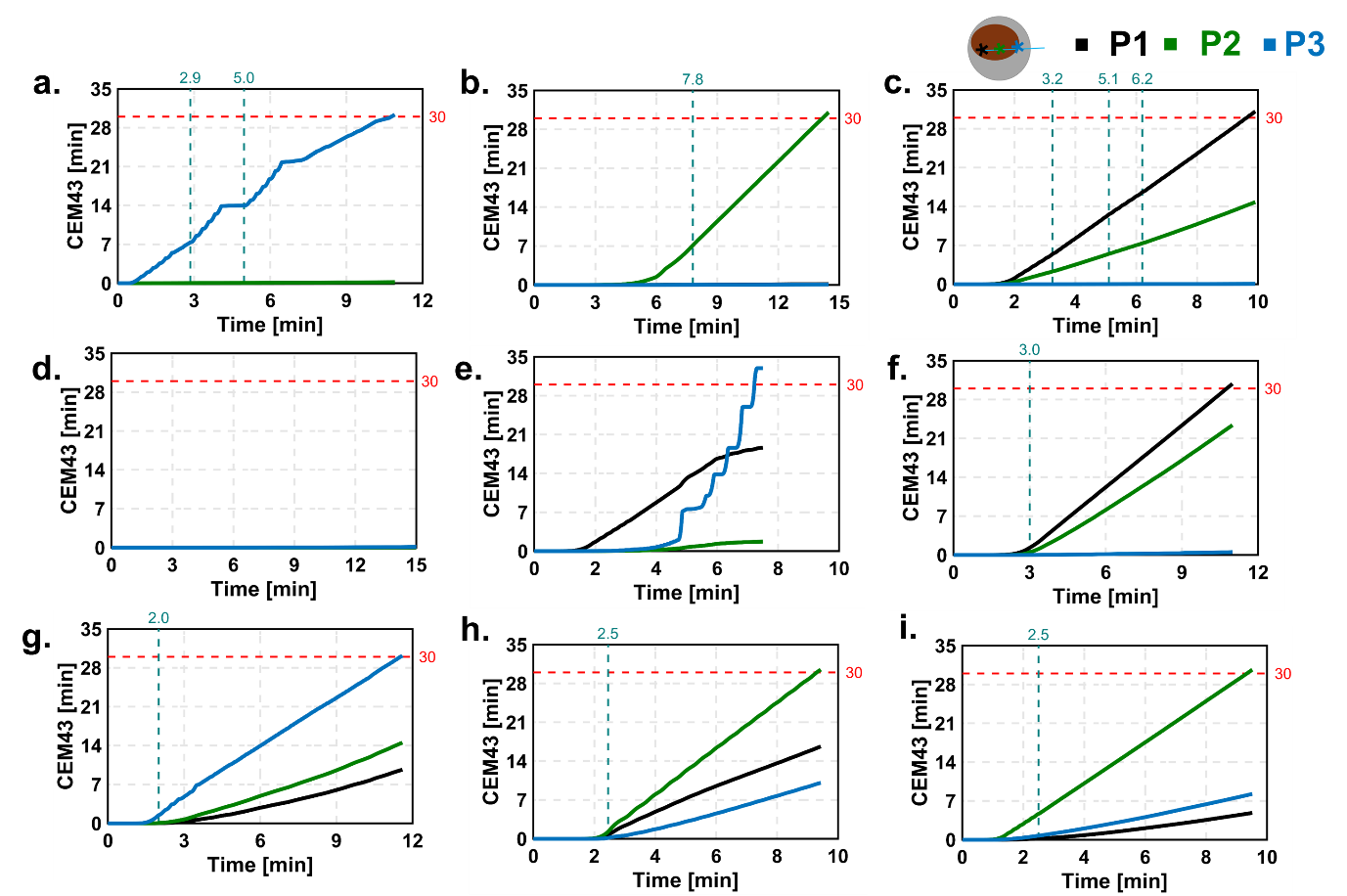
**

**Figure S3: Thermal dose (CEM43) evolution during the treatment.** **a.** Mouse 1; **b.** Mouse 2; **c.** Mouse 3; **d.** Mouse 4; **e.** Mouse 5; **f.** Mouse 6; **g.** Mouse 7; **h.** Mouse 8; **i.** Mouse 9.
